# Natural Deep Eutectic Solvent (NADES) Extraction Improves Polyphenol Yield and Antioxidant Activity of Wild Thyme (*Thymus serpyllum* L.) Extracts

**DOI:** 10.3390/molecules27051508

**Published:** 2022-02-23

**Authors:** Branimir Pavlić, Živan Mrkonjić, Nemanja Teslić, Aleksandra Cvetanović Kljakić, Milica Pojić, Anamarija Mandić, Alena Stupar, Filipa Santos, Ana Rita C. Duarte, Aleksandra Mišan

**Affiliations:** 1 Faculty of Technology, University of Novi Sad, Blvd. cara Lazara 1, 21000 Novi Sad, Serbia; zivan_mrkonjic@hotmail.com (Ž.M.); a.c.istrazivac@gmail.com (A.C.K.); 2 Institute of Food Technology, University of Novi Sad, Blvd. cara Lazara 1, 21000 Novi Sad, Serbia; nemanja.teslic@fins.uns.ac.rs (N.T.); milica.pojic@fins.uns.ac.rs (M.P.); anamarija.mandic@fins.uns.ac.rs (A.M.); alena.tomsik@fins.uns.ac.rs (A.S.); 3 LAQV, REQUIMTE, Departamento de Química, Nova School of Science and Technology, 2829-516 Caparica, Portugal; mfca.santos@campus.fct.unl.pt (F.S.); ard08968@fct.unl.pt (A.R.C.D.)

**Keywords:** *Thymus serpyllum* L., NADES, polyphenols, antioxidant activity, RSM optimization

## Abstract

Wild thyme (*Thymus serpyllum* L.) herbal dust has been recognized as a potential underutilized resource for the recovery of antioxidants. The aim of this paper was to optimize natural deep eutectic solvent (NADES) extraction of polyphenols to obtain improved antioxidant activity of extracts determined by selected in vitro assays (DPPH, FRAP, and ABTS). Twenty different NADES systems were investigated in the first step of the screening of the extraction solvent and l-proline (Pro)–glycerine (Gly) based solvents provided the best results. Preliminary experiments organized by 2^5−1^ fractional factorial design narrowed down the number of extraction factors from five (temperature, extraction time, NADES type, water content and L/S ratio) to three and determined their experimental domain for the final step. A face-centered central composite design with temperature (40–55–70 °C), extraction time (60–120–180 min) and L/S ratio (10–20–30 g NADES/g sample) was applied for influence analysis and process optimization. Multi-response optimization suggested a temperature of 65 °C, time of extraction of 180 min and L/S ratio of 28 g NADES/g DW as optimal extraction parameters. Experimental validation confirmed good agreement between experimental and predicted results in the extract obtained at optimal conditions and the interactions in the most suitable NADES (N16; Pro–Gly–H_2_O; 1:2:1) were confirmed by the ^1^H-NMR.

## 1. Introduction

Wild thyme (*Thymus serpyllum* L.) is an aromatic plant belonging to the Lamiaceae family. This herb is widely used thanks to its biological activities, such as antiseptic, antitussive, analgesic, anthelmintic, diaphoretic, expectorant, spasmolytic, carminative and diuretic activities [1,2]. Traditionally, it has been most frequently used in problems related to digestive, respiratory and urogenital tracts [1]. The reason for all the aforementioned possibilities for use is the high content of polyphenols, which represent compounds of interest in the present research.

Nowadays, a lot of focus is placed on the valorization of industrial waste, using more effective energy and solvents recognized as safe, which is underlined with the main principles of green extraction [3,4,5]. Furthermore, designing the production line with a minimal number of processing steps and obtaining a safe, non-denatured and biodegradable extract without concomitants as a final product lead to the fact that green extraction processes of natural products present a desirable approach for the isolation of antioxidants [3,6]. The most prominent representatives of green extraction techniques are extractions assisted with microwaves, ultrasounds and pulsed electric fields, as well as extractions based on using solvents at sub- and supercritical level and natural deep eutectic solvents (NADES), with the possibility of combining them.

In addition, conventional solid-liquid, ultrasound-assisted (UAE) and pressurized liquid extractions (PLE) have proven to be a great approach for antioxidant recovery from *T. serpyllum* herbal dust, using ethanol as an extraction solvent [7,8]. On the other hand, considering the fact that conventional solvents could be flammable, volatile and toxic, their daily use can become problematic for humans and generally for environmental persistence and/or photochemical ozone creation [9]. There is also a growing incentive to research alternative solvents that would retain the technological properties of organic solvents and at the same time have a favorable impact on human health and the environment. In recent years, the development of alternative solvents has focused on NADES. They have shown the greatest potential in the field of green chemistry, due to the fact that they are abundant, inexpensive, recyclable and attractive for food, cosmetic and pharmaceutical applications [10,11,12]. So far, many studies have successfully conducted NADES extraction in order to obtain high-quality extracts from various plants, such as from native Greek medicinal plants [10], olive pomace [13], lemon verbena [14], peppermint and lemon balm [15], sour cherry pomace [16] and blueberry [17]. Another great feature of NADES is its potential biological activity, bioavailability and the possibility of an untitled number of solvent combinations for their preparation [17,18]. Therefore, NADES extraction represents an innovative technique that has broadened curiosity among scientific circles and has already proven great potential in the field of extraction and isolation of bioactive compounds, as well as their application in various industry branches. Aerial parts of wild thyme (*Thymi serpylli herba*) were suggested as official preparation of expectorants [19]; therefore, the application of wild thyme extracts obtained by NADES extraction could be considered in expectorant syrup formulations.

The main objective of this study was to valorize *T. serpyllum* herbal dust as raw material for antioxidant recovery using NADES extraction. The study consisted of three phases, where in the initial one, according to total phenol content (TP) and antioxidant properties of obtained extracts, two of the twenty different NADES systems were selected. The second phase encompassed choosing the most influential parameters and to find their domain, by the evaluation of extraction parameters (temperature, extraction time, liquid-solid (L/S) ratio, NADES type and water content in the solvent) using 2^5−1^ fractional factorial design. Finally, the main experiments were performed using response surface methodology (RSM) in order to conduct the optimization of polyphenol recovery from *T. serpyllum* using desirability function with Y, TP, total flavonoid content (TF) and antioxidant activity determined toward DPPH, ferric ion reducing antioxidant power (FRAP) and ABTS assays as target responses. Finally, validation was done and the structure and nature of optimized NADES was determined by nuclear magnetic resonance spectroscopy (NMR).

## 2. Results and Discussion

### 2.1. Screening of the Extraction Solvent

Natural deep eutectic solvents (NADES) have been exhaustively applied for the recovery of polyphenols recently, although no application on the recovery of wild thyme (*Thymus serpyllum* L.) polyphenols has been found in recent literature. Fernandez et al. [20] published an in-depth review of NADES application on the isolation of different compounds from natural resources, among them polyphenols, where it could be observed that a huge variety of NADES could be selected as optimal solvents depending on the physicochemical properties of target molecules and plant material characteristics. Therefore, the screening of the extraction solvent was performed as an initial step in the case study of extraction of wild thyme bioactives. Twenty NADES mixtures, given in Table 1, were prepared for that purpose. All other extraction parameters were held constant in order to obtain information about the influence of NADES type using the OFAT approach, while TP and scavenging capacity toward DPPH radicals were measured responses.

**Figure 1:**
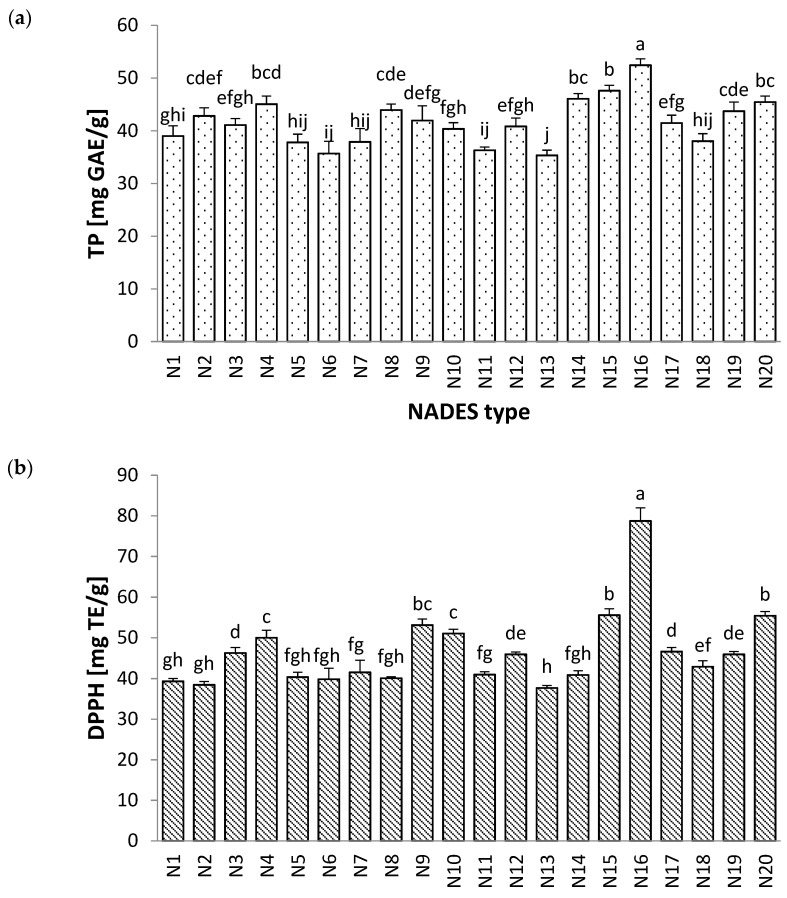
The effect of applied NADES on (**a**) total phenol content and (**b**) antioxidant activity toward DPPH radicals in wild thyme extracts. Results were expressed as mean ± standard deviation (SD) and different letters represent statistically significant differences (*p* < 0.05) according to Tukey’s test.

TP in wild thyme extracts was in the range of 35.33 to 52.43 mg GAE/g. It could be observed that NADES based on l-proline (N15 and N16) provided the highest TP values (47.61 and 52.43 mg GAE/g) (Figure 1a). N15 was based on l-proline and lactic acid (1:2), while N16 was made of l-proline, glycerine and water (1:2:1). Water was an integral compound in the N16 mixture, which significantly improved TP (*p* < 0.05). The range of results for the scavenging capacity of DPPH radicals was 37.79–78.73 mg TE/g (Figure 1b). Similarly, extracts obtained by N15 and N16 exhibited the highest activity (55.55 and 78.73 mg TE/g), even though no significant difference (*p* > 0.05) was observed between the samples obtained by N15, N9 (ChCl–Gly; 1:2) and N20 (PD–ChCl–H_2_O; 1:1:1). Good Pearson’s correlation was observed between TP and DPPH data (*r* = 0.755; *p* < 0.05), suggesting that polyphenols were the main compounds responsible for antioxidant activities in *T. serpyllum* extracts.

There are several examples where Pro–Gly-based NADES were the best solvent mixture for the recovery of polyphenolic compounds. This could be explained by the significant affinity of polyphenols for extended proteins and peptides that contain a high proportion of Pro residues in their sequences [21]. According to Nam et al. [22], NADES with Pro–Gly (2:5) was found to be the best solvent for the isolation of flavonoids (quercetin, kaempferol and isorhamnetin glycosides) from flowers of *Sophora japonica*. l-proline-based NADES mixtures with organic acids were found to be very useful in the isolation of different polyphenol groups. Mansinhos et al. [23] performed screening of 10 NADES systems for the recovery of bioactives from *Lavandula pedunculata* subsp. *lusitanica* and observed that the highest TP and antioxidant activities determined by different in vitro assays were obtained by Pro–LA (1:1). Huang et al. [24] performed comprehensive studies focused on the investigation of 25 different NADES mixtures on the isolation of puerarin flavones from the root of *Pueraria lobata* and observed that the utilization of Pro–MA solvent improves the yield of target compounds, as well as their oral bioavailability.

Despite the enormous potential of NADES solvents, their high viscosity has been recognized as the main disadvantage. Dai et al. [25] reported that the addition of a small amount of water into NADES solvents could reduce their viscosity and increase their conductivity. According to the results from Figure 1, Pro–Gly with a higher content of water also provided significantly higher TP and DPPH. Furthermore, it has been reported that NADES based on proline could improve the solubility and bioavailability of certain flavonoids, such as rutin [26]. Taking all this into consideration, N15 and N16 solvents, both based on Pro–Gly, were selected for further research.

### 2.2. Preliminary Study

Besides the NADES system, the extraction of bioactive compounds could be affected by several different factors that should be optimized for each case study. Guo et al. [27] used the Plackett–Burman design for the preliminary study to narrow down the number of factors affecting the NADES extraction of mulberry anthocyanins from seven to three. These authors identified L/S ratio, homogenization time, homogenization speed, negative pressure, extraction time, temperature and number of cycles as factors for preliminary study. On the other hand, Shikov et al. [28] used the Plackett–Burman design with five factors (particle size, extraction time, temperature, extraction modulus and water content) for a preliminary study of NADES extraction of *Rhodiola rosea* L. Taking this into consideration, we organized a preliminary study using 2^5−1^ fractional factorial design with five factors. NADES type was selected as a categorical variable, while temperature, extraction time, S/L ratio and water content were numerical variables. The aim of the preliminary study was to determine the most influential NADES extraction parameters affecting TP and DPPH in order to obtain an experimental design for the optimization study.

The experimentally observed values of target responses (TP and DPPH) obtained in the 16 runs of the preliminary study are given in Table 1. TP ranged from 43.31 to 64.24 mg GAE/g, suggesting that TP could be improved by variation of other extraction parameters. This was a similar case with DPPH since experimental values were in the range of 37.59–91.16 mg TE/g. The lowest and highest values of TP and DPPH were observed at the same experimental runs (run 13 and run 12, respectively), confirming the positive linear correlation observed between TP and DPPH in the first step of this research.

**Figure 2:**
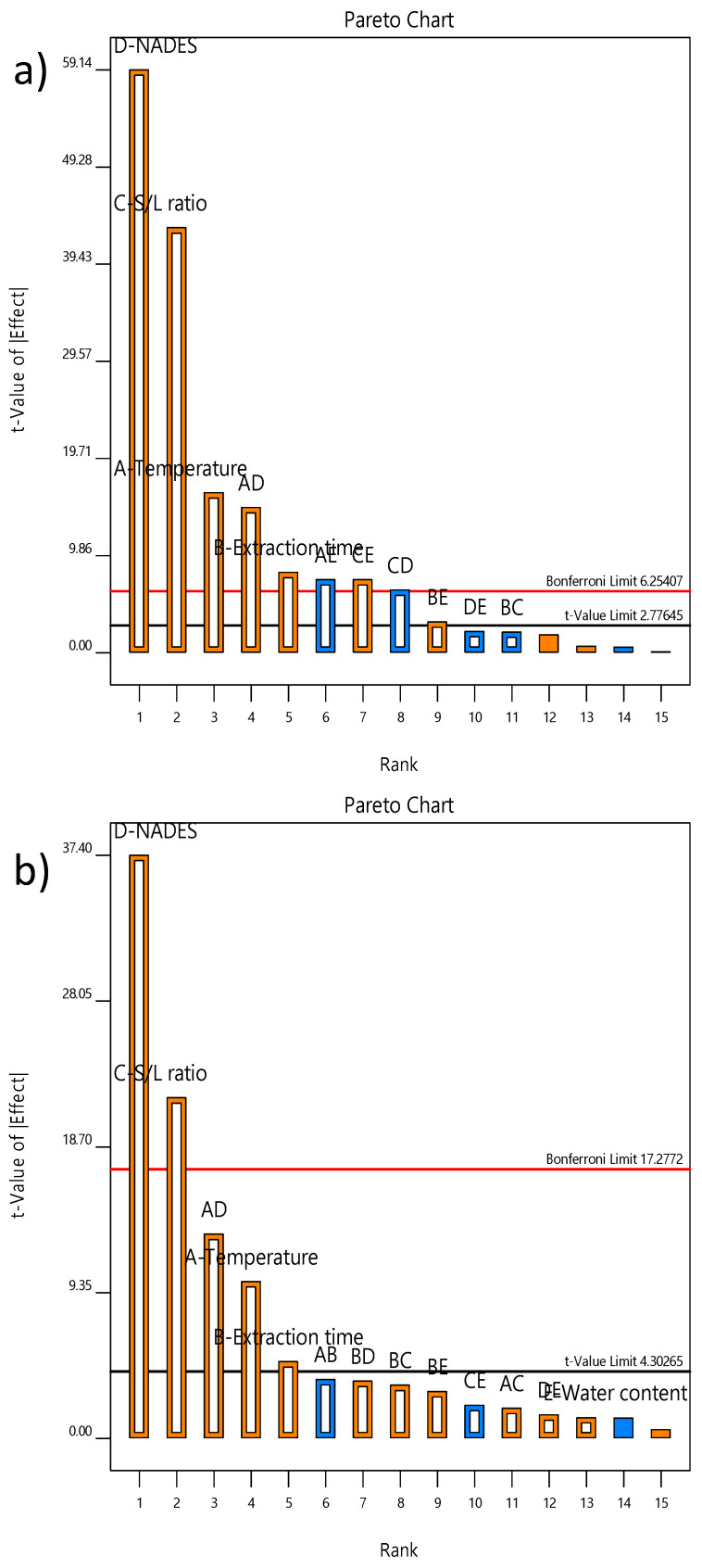
Pareto chart exhibiting effects of temperature (A), extraction time (B), L/S ratio (C), NADES (D) and water content (E) on (**a**) TP and (**b**) DPPH.

The TP and DPPH data were fitted to an interaction model given in Equation (1) and influence analysis was determined using Pareto charts. It is evident that NADES type exhibited the strongest impact on the target responses, while its influence was followed by the L/S ratio, temperature and extraction time. The influence of these four factors was significant according to t-values, while the impact of water content was insignificant (Figure 2). It should be highlighted that temperature–NADES type interaction also exhibited a significant influence on both responses. Since NADES exhibited a significant impact on TP and DPPH and since notably higher values of target responses were obtained using N16, this solvent system (Pro–Gly–H_2_O; 1:2:1) was selected for the optimization study. Since water content in the NADES mixture did not exhibit a significant effect on TP and DPPH, water content was fixed at 20% to be used in further experiments. Besides downsizing the number of extraction factors from five to three, the preliminary study was used for the determination of the experimental domain to be used in further steps. The influence of independent variables can be observed in Figure S1 (Supplementary Materials). A positive linear influence could be observed for temperature, extraction time and L/S ratio (Figure S1). Several recent NADES applications used different approaches to organize the optimization study. Doldolova et al. [29] investigated NADES extraction of antioxidants from turmeric and performed initial screening of five NADES solvents. Further, they organized the RSM study in a similar range of temperature (45–60–75 °C); however, they combined NADES extraction with microwave-assisted extraction (MAE) and the range of the extraction time and L/S ratio were 5–30 min and 10–20 mL/g. Similarly, screening of the NADES type was followed by RSM optimization with extraction time (40–100 °C), water content in NADES (10–50%) and molar ratio between LA and ChCl (1–5 mol/mol) in the extraction of polyphenols from *Helichrysum arenarium* L. [30].

Taking into consideration trends of the influence of the extraction parameters obtained in the preliminary study and recent literature data on NADES extraction, the experimental plan for the RSM optimization study was generated with experimental domain for temperature, extraction time and L/S ratio set at 40–55–70 °C, 60–120–180 min and 10–20–30 g NADES/g of plant sample, respectively, while NADES type (N16) and water content (20%) were held constant in this step.

### 2.3. Optimization Study

#### 2.3.1. Accuracy of Fit and Influence Analysis

Experimental results of investigated responses (TP, TF, DPPH, FRAP and ABTS) obtained under different sets of NADES extraction parameters (temperature, extraction time and L/S ratio) using a face-centered central composite experimental design are given in Table 2. Data was fitted to a quadratic polynomial model (Equation (2)), while ANOVA (F-test) and descriptive statistics (R^2^ and CV) were applied in order to determine goodness of fit (Table 3). Generally, high values of R^2^ were observed for TP, TF and DPPH (0.922, 0.905 and 0.984, respectively), while moderately high values were obtained in the case of FRAP and ABTS (0.832 and 0.803), suggesting the accordance between the model and experimental results. Moreover, TP and DPPH results were followed by negligible CV (4.72 and 3.69%), which points out proper reproducibility of the developed model systems. Moderately high CV values for TF, FRAP and ABTS (17.24, 12.88 and 13.48%, respectively) suggest that higher dispersion of the data could occur. Furthermore, thorough information about model fitness could be obtained by the lack-of-fit testing since insignificant lack-of-fit confirms the assumption of the constant variance, which means that variance is a model-independent measure of the pure error [31]. It was shown that the quadratic model provides adequate representation of experimental data according to statistically insignificant *p*-values for the lack-of-fit (*p* > 0.05) for the TP, DPPH, FRAP and ABTS. Slight disagreement in statistical parameters for certain responses suggests that experimental validation must be performed in order to confirm the developed mathematical models. Several other studies showed that RSM could be adequately used for optimization of NADES-based extractions, i.e., NADES extraction combined with UAE for isolation of *Helichrysum arenarium* polyphenols [30], NADES extraction combined with MAE for isolation of turmeric antioxidants [29], recovery of mulberry anthocyanins [27] and wine lees anthocyanins [32], and isolation of TP from *Hibiscus sabdariffa* [33].

**Figure 3:**
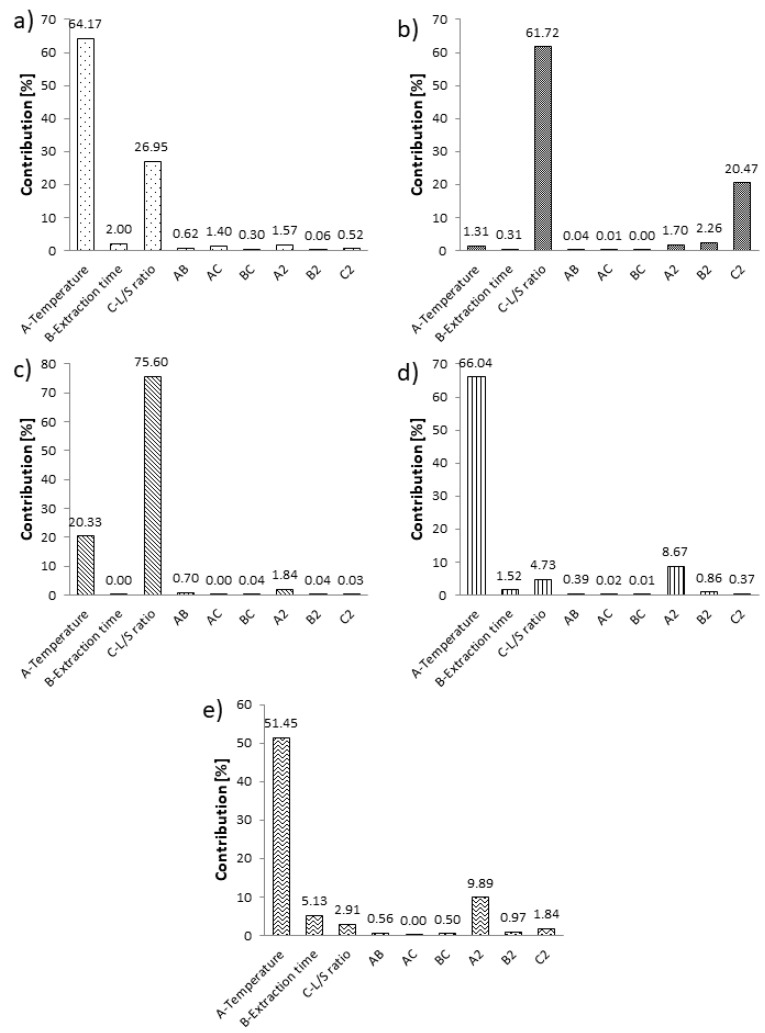
Contribution plots for the linear, interaction and quadratic terms on (**a**) TP, (**b**) TF, (**c**) DPPH, (**d**) FRAP and (**e**) ABTS.

Contribution analysis pointed out that the linear term of temperature was the most prominent factor (*p* < 0.05) affecting the TP, DPPH, FRAP and ABTS with a contribution higher than 50% (Figure 3). The quadratic term of temperature exhibited a significant effect on DPPH, while its effect on FRAP and ABTS was moderate (*p* < 0.1) (Table S1). Temperature was also found to be the most influential parameter in an RSM study focused on NADES combined with MAE for the isolation of bioactive compounds from turmeric [29]. On the other hand, L/S ratio was the most dominant NADES extraction parameter that affected TF and DPPH with a contribution above 60% (Figure 3), while the quadratic term of this factor was statistically significant (*p* < 0.05) only in the case of TF with a 20.47% contribution. It could be observed that neither the extraction time terms nor any interaction caused a significant impact on any response (Table S1). According to Guo et al. [27], L/S ratio exhibited significant, while the extraction time effect was insignificant in the RSM study focused on the NADES extraction of mulberry anthocyanins.

Contribution analysis pointed out that the linear term of temperature was the most prominent factor (*p* < 0.05) affecting TP, DPPH, FRAP and ABTS with a contribution higher than 50% (Figure 3). The quadratic term of temperature exhibited a significant effect on DPPH.

For the calculation of regression coefficients in polynomial equation, the method of least square was used and gave the predictive model equations that are as follows:

$$\mathrm{TP}=60.84\;7.31A+1.29B+4.73C+0.81\mathrm{AB}+1.21\mathrm{AC}+0.55\mathrm{BC}-2.19A^{2}+0.43B^{2}-1.25C^{2}$$

$$\mathrm{TF}=22.23-1.05A-0.51B+7.22C-0.20\mathrm{AB}+0.11\mathrm{AC}-0.07\mathrm{BC}-2.29A^{2}+2.64B^{2}-7.95C^{2}$$

$$\mathrm{DPPH}=146.38+17.36A+0.10B+33.49C-3.60\mathrm{AB}-0.16\mathrm{AC}+0.88\mathrm{BC}-9.99A^{2}-1.44B^{2}+1.17C^{2}$$

$$\mathrm{FRAP}=37.55+7.40A+1.12B+1.98C+0.64\mathrm{AB}+0.13\mathrm{AC}-0.09\mathrm{BC}-5.13A^{2}-1.61B^{2}-1.05C^{2}$$

$$\mathrm{ABTS}=124.42+20.12A+6.37B+4.79C+2.35\mathrm{AB}+0.22\mathrm{AC}-2.22\mathrm{BC}-16.91A^{2}-5.30B^{2}-7.30C^{2}$$

#### 2.3.2. Polyphenol Content, Antioxidant Activity and Effect of NADES Extraction Parameters

To the best of our knowledge, NADES extraction was not previously optimized for *T. serpyllum*. TP varied between 47.38 and 71.43 mg GAE/g (Table 2) depending on the different set of NADES extraction conditions. The highest TP was observed in run 17, which was obtained at high levels of independent extraction factors (70 °C, 180 min and 30 g NADES/g plant material). On the other hand, recent work suggested that extraction of polyphenols from wild thyme herbal dust was optimized by other techniques, such as conventional solid-liquid extraction, ultrasound-extraction (UAE) and pressurized-liquid extraction (PLE) [7,8]. According to Mrkonjic et al. [7], ethanol concentration was an optimized factor for conventional extraction of *T. serpyllum* herbal dust and the highest TP observed was 17.35 mg GAE/g, while 43.88 mg GAE/g of TP was obtained by UAE at an optimal set of temperature, extraction time and ethanol concentration. In other work, the PLE of wild thyme herbal dust was optimized in two stages (preliminary design and RSM study), and 67.46 mg GAE/g of TP was obtained at an optimal set of PLE conditions [8]. Experiments in this work were performed with the same raw material as was used in the aforementioned references, which highlights the tremendous improvement in TP obtained by NADES extraction compared to traditional extraction and UAE, as well as considerable improvement compared to PLE. In the case of TF, experimentally measured values ranged from 4.82 to 23.54 mg CE/g and it could be generally observed that high TF values were observed at the experimental runs with high TP. TF in wild thyme extracts obtained by UAE and maceration was approximately 50% of TP; however, TP results in these works were exhibited as mg CE per liter of liquid extracts [34,35], which makes it unsuitable for comparison. However, it could be observed that the TP/TF ratio obtained in this work was considerably lower (Table 2) compared to the aforementioned research studies.

Fast in vitro assays were selected as model systems for the determination of antioxidant activity. Experimentally observed values obtained by the DPPH, FRAP and ABTS assays were in the following ranges: 78.63–188.01 mg TE/g, 19.85–44.70 mg Fe^2+^/g and 70.81–147.87 mg TE/g, respectively. It could be observed that the lowest values obtained by all three assays were obtained in run 2 at a lower level of all three factors (40 °C, 60 min and 10 g NADES/g DW). On the other hand, the highest activity toward DPPH radicals was observed in run 17, the same extract where the highest TP was obtained, suggesting that polyphenols were the main compounds responsible for antioxidant activity. In vitro antioxidant activity in wild thyme extracts obtained by various extraction techniques (conventional solid-liquid, UAE and PLE) was previously investigated elsewhere and the results were expressed as mM equivalents per 100 g plant material [7,8]. However, when these results are calculated on the same units as was given in this work, the radical scavenging capacity of *T. serpyllum* extracts obtained by conventional solid-liquid extraction, UAE and PLE were 34.37, 63.35 and 79.42 mg TE/g and it could be noted that NADES extraction provides tremendous upgrade on antioxidant capacity of wild time extracts in comparison with other traditional and emerging extraction techniques (Table 2). On the other hand, FRAP results were 43.18 and 47.96 mg Fe^2+^/g and ABTS results were 131.17 and 173.79 mg TE/g observed in wild thyme extracts obtained by UAE and PLE techniques, respectively [7,8]. It could be noted that NADES extraction provides very similar results of reducing power and scavenging capacity of ABTS^+^ radicals as other emerging extraction techniques.

**Figure 4:**
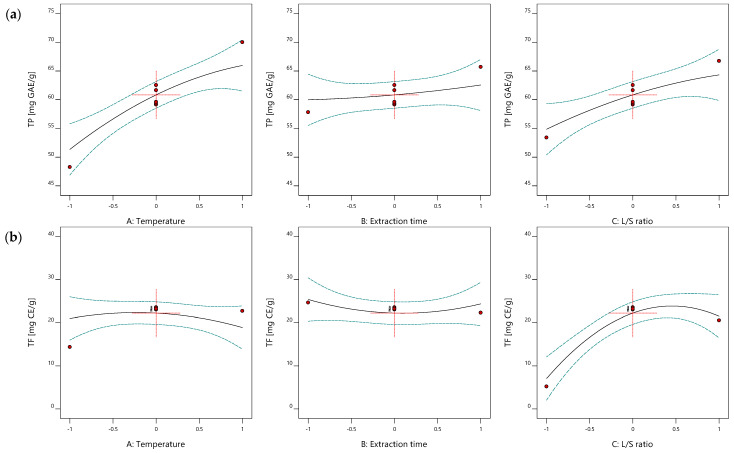
The effect of NADES extraction parameters (temperature, extraction time and L/S ratio) on (**a**) TP and (**b**) TF.

Since no significant interactions were observed (Table S1), impacts of NADES extraction parameters were presented in one-factor graphs given in Figure 4.

Temperature has often been considered the most important extraction factor, despite the applied technique. The linear term of temperature exhibited a strong positive effect on TP, FRAP and ABTS, which could be explained by different phenomena occurring within the process. Increased temperature decreases surface tension and the viscosity of the solvents, which is particularly important for viscous fluids, such as NADES mixtures. This will result in the wetting of the sample and easier penetration of the solvent in the matrix [36]. At the same time, elevated temperature will ease the desorption and dissolution of the target compounds by preventing adhesion to the solid matrix weakening physicochemical interactions, such as van der Waals forces, hydrogen bonding, dipole moment and electrostatic interactions [29]. The positive impact of the linear term of temperature was often reported in other works focused on NADES-based processes optimized by RSM [33]. However, elevated temperature could cause chemical degradation of either NADES or target compounds and some reports suggested yellowing of the solvent, which is considered to be degradation of the NADES at temperature above 75 °C [29]. Therefore, a high level of temperature would be optimal for the maximized recovery of polyphenols (TP) and antioxidant activity Figure 4a and Figure S2), while no significant effect of temperature on TF was observed (Figure 4b).

The temperature effect was followed by the significant positive impact of L/S ratio which is in accordance with mass transfer laws. A higher L/S ratio will provide a better driving force due to the higher concentration gradient between NADES and the plant matrix [29]. However, an insufficient L/S ratio could cause various equilibriums in the process and consequently higher resistance to a mass transfer [37]. The impact of L/S ratio was in accordance with other case studies where NADES extraction was optimized using experimental design [27,33]. However, a tremendous L/S ratio would diminish the content of the target compounds in the extracts obtained by NADES, which would limit their utilization in other food, cosmetics or pharmaceutical products. Therefore, a high level of L/S ratio (30 g NADES/g DW) would be optimal for the recovery of wild thyme antioxidants Figure 4 and Figure S2), which is the range that was generally used in similar NADES-RSM studies [27]. Furthermore, a significant effect of the quadratic term of L/S ratio on TF can be clearly observed in Figure 4b.

#### 2.3.3. NMR Characterization of the Optimal NADES

**Figure 5:**
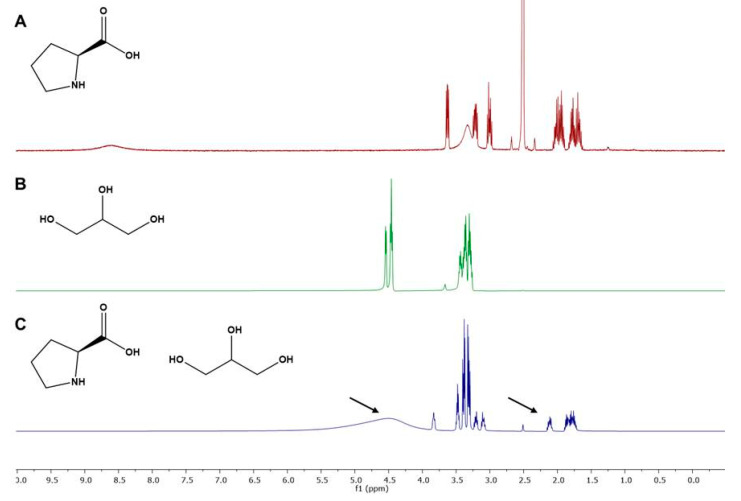
^1^H-NMR spectra of (**A**) l-proline, (**B**) glycerine and (**C**) N16 (20% *w/w* of water).

The NMR experiments were performed in individual components used for the preparation of optimal NADES (N16; Pro–Gly–H_2_O; 1:2:1) and a mixture used for the extraction. ^1^H spectra were overlaid to facilitate the observation of differences between chemical shifts of the individual components and after NADES formation (Figure 5). It is observed that all the groups in the NADES formulation suffer from a slight deviation in the chemical shift, which suggests a different spatial distribution of the molecules. Although in NOESY we only observe signals in the -NH group of l-proline and -CH_2_ of glycerine, we can confirm a proximity between the two components of NADES and predict that there are interactions occurring in -OH and -NH groups from glycerine and l-proline.

#### 2.3.4. Optimization and Validation

In order to simultaneously maximize polyphenol content (TP and TF) and antioxidant activity (DPPH, FRAP and ABTS) and to determine the best set of NADES extraction parameters, desirability function was used and its value was 0.887. Multi-response optimization provided the following optimal conditions: temperature of 65 °C, extraction time of 180 min and L/S ratio of 28 g NADES/g DW. Predicted and experimental values of investigated responses are presented in Table 4. After experimental verification at optimal conditions, the agreement between experimental and predicted values was very good. Minor disagreement was observed in the case of TF and ABTS since experimentally observed values were slightly lower compared to calculated predictions (Table 4). This leads to the conclusion that applied quadratic models are validated and could be used for point prediction within the investigated experimental domain. It should be highlighted that performed optimization in three steps provided an increase in TP and DPPH for approximately 26% and 56%, respectively, compared to the highest values obtained in the first step (screening of the extraction solvent). Since it has been previously confirmed that NADES extraction provides a significant upgrade in the recovery of polyphenols from wild thyme compared to conventional extraction, UAE [7] and PLE [8], it could be concluded that NADES extraction is an efficient method and emerging approach for the recovery of *T. serpyllum* antioxidants.

HPLC analysis of major polyphenols was performed on NADES extract obtained under optimal conditions, and results are given in Table 5. Rosmarinic acid was the predominant compound present in wild thyme extract with particularly high content (524.18 mg/100 g), which is in accordance with previously published data on wild thyme extracts [2,34]. On the other hand, luteolin, epicatechin and quercetin were major flavonoids quantified in this sample. Previous studies confirmed that all compounds quantified in this work were identified in wild thyme extracts obtained by UAE [7] and PLE [8]. HPLC results showed that a particularly high content of phenolic acids and flavonoids could be obtained by NADES extraction.

## 3. Materials and Methods

### 3.1. Plant Material

*T. serpyllum* L. used as a raw material in the present study represents a by-product from filter-tea production that was kindly donated by the factory Macval D.O.O. from Novi Sad (Serbia). Plant material was harvested and processed in 2017. Since its mean particle size is less than 0.315 mm, it cannot be packed in filter bags; therefore, it is considered industrial waste. Dried herbal dust was stored in paper bags at 20 °C.

### 3.2. Chemicals

From Sigma-Aldrich (Steinheim, Germany), the following reagents were obtained: Folin–Ciocalteu reagent, Trolox, gallic acid, 2,2-diphenyl-1-picrylhydrazyl (DPPH), 2,4,6-tris(2-pyridyl)-*s*-triazine (≥99.0%) (TPTZ), glucose, sucrose, choline chloride, glycerin, fructose, lactic acid (85% purity with 15% water), l-proline, 1,2-propanediol and citric acid. 2,2′-Azino-bis(3-ethylbenzothiazoline-6-sulfonic acid) diammoniumsalt (98%) was purchased from J&K, Scientific Ltd., Beijing, China. l(+)-tartaric acid and malic acid were obtained from Scharlab (Barcelona, Spain), while anhydrous betaine was purchased from Tokyo Chemical Industry (Tokyo, Japan). Additionally, sodium carbonate anhydrous and ferric chloride hexahydrate were supplied from Centrohem, Stara Pazova, Serbia. Acetic acid (99.8%) and potassium peroxydisulfate were purchased from Lach-Ner, Neratovice, Czech Republic, while sodium acetate anhydrous was purchased from Kemika, Zagreb, Croatia. Polyphenol standards used for HPLC analysis (gallic acid, caffeic acid, epicatechin, rosmarinic acid, luteolin and quercetin) were all purchased from Sigma-Aldrich (Steinheim, Germany). Ultrapure water was obtained by a Milli-Q Plus system (EMD Millipore, Billerica, MA, USA). All other chemicals were of analytical reagent grade.

### 3.3. NADES Preparation

All NADES used in the present study are composed of organic acids and polyols as hydrogen-bond donors and hydrogen-bond acceptors with different molar ratios (Table 6). According to Ekaterina et al. [38], NADES could be efficiently formulated for the recovery of both hydrophilic and lipophilic compounds. The selection and formulation of NADES used in this work was based on applications of NADES extraction for recovery of polyphenols from different plant matrices found in recent literature [39,40,41,42]. In total, twenty different NADES were selected for the screening study, and they were prepared in a water bath at 80 °C placed on a magnetic stirrer hot plate. Mixing lasted approximately 10 min until stable transparent liquid was formed. All created NADES were stable at room temperature (≈20 °C) up to more than seven days. Final water content in NADES was mathematically calculated (20 or 25%) and accordingly adjusted for each extraction run according to our previous study [39]. Initial water content in chemicals and reagents was also taken into consideration for calculation.

### 3.4. NADES Extraction and Experimental Plan

NADES extraction of wild thyme polyphenols was performed in three steps: (1) screening of extraction solvent, (2) preliminary study and (3) response surface methodology (RSM) optimization. The experimental plan is given in Table 7.

Experiments for the screening of extraction solvent were performed in a water bath at 50 ± 1 °C placed on a magnetic stirrer hot plate with thermocouple for temperature regulation. Solvent (NADES: N1–N20) and plant matrix (0.05 g) were placed together with a small magnet into a glass extraction vial with a 1:20 (m/m) sample to solvent ratio. The vial was tightly closed with a cap and immersed in the water bath for 60 min. Water (4 mL) was added after the extraction to ease separation of solid and liquid phases, and samples were centrifuged for 15 min at 4000 rpm. The supernatant was separated from solid plant residue and stored in a fridge at 4 °C until the analysis of total phenol content (TP) and antioxidant activity toward DPPH radicals.

Since N15 and N16 were the solvents that provided the highest total phenol content and antioxidant activity, they were selected as categorical variables in the preliminary study. Fractional 2^5−1^ factorial was used for that purpose, with a total of 16 experimental runs. Numerical factors at two levels were temperature (50 and 60 °C), extraction time (60 and 120 min), sample to solvent ratio (1:10 and 1:20 m/m) and water content (20 and 25%), while TP and DPPH were target responses. In order to determine the impact of NADES parameters on target responses, the linear model given by Equation (1) was used.

(1) $$Y=\beta_{0}+\sum_{i=1}^{5}\beta_{i}X_{i}+\sum_{i=1}^{4}\sum_{j=i+1}^{5}\beta_{\mathrm{ij}}X_{i}X_{j}\;$$

*Y* represents the response variable, β_0_ the intercept, β_i_ the linear regression coefficient, β_ij_ the regression coefficients for cross-product terms and X_i_ and X_j_ the independent variables affecting the response.

After screening, three of the five most influential parameters were selected, which were further used in face-centered central composite experimental design (CCD) with response surface methodology (RSM). The impact of temperature (40, 55 and 70 °C), extraction time (60, 120 and 180 min), and sample to solvent ratio (1:10, 1:20 and 1:30 m/m) were used as independent variables. Optimal extraction conditions were determined considering TP, as well as antioxidant activity parameters obtained by DPPH, ABTS and FRAP assays, while selection of optimal conditions were based on desirability function (*D*) [43]. Results were fitted to a second-order polynomial model (Equation (2)).

(2) $$Y={\;\beta }_{0}+\sum_{i=1}^{3}\beta_{i}X_{i}+\sum_{i=1}^{3}\beta_{\mathrm{ii}}X_{i}^{2}+\sum_{i=1}^{2}\sum_{j=i+1}^{3}\beta_{\mathrm{ij}}X_{i}X_{j}\;$$

*Y* represents the response variable, X_i_ and X_j_ are the independent variables affecting the response, and β_0_, β_i_, β_ii_, and β_ij_ are the regression coefficients for intercept, linear, quadratic and cross-product terms.

### 3.5. Polyphenol Analysis

#### 3.5.1. Total Phenols Content

For the determination of TP in extracts, the Folin–Ciocalteu assay was used [44]. Using a spectrophotometer (Jenway, model 6300, Staffordshire, UK), absorbances were recorded at 750 nm, and all experiments were performed in triplicate. Mean values of the total phenols of obtained extracts were expressed as mg of gallic acid equivalents (GAE) per g of sample dry weight.

#### 3.5.2. Total Flavonoids Content

Determination of TF was done using an aluminum chloride colorimetric assay [45]. Catechin was used as the standard for the preparation of the calibration curve, and absorbances were measured at 510 nm. Results were expressed as mg of catechin equivalents (CE) per g DW.

#### 3.5.3. HPLC Analysis of Major Polyphenols

For identification and quantification of individual phenolic compounds, the HPLC method, previously published by Mišan et al. [46], was done on an Agilent 1200 series device with a diode array detector (DAD). HPLC analysis was performed on a liquid chromatograph (Agilent 1200 series, Agilent, Santa Clara, CA, USA), equipped with a diode array detector (DAD) and Eclipse XDB-C18, 1.8 μm, 4.6 × 50 mm column. Solvents A (methanol) and B (1% formic acid in water (*v/v*)) were used as mobile phases, with a flow rate of 1 mL/min. A solvent gradient was performed by varying the proportion of solvent A to solvent B as follows: at start 10% A; 0–10 min, 10–25% A; 10–20 min, 25–60% A; 20–30 min, 60–70% A. The column temperature was set at 30 °C, while the injection volume was 5 μL. Optimized *T. serpyllum* extract was properly diluted with a mixture of mobile phases (A:B = 10:90%; *v/v*), filtered through a syringe filter (RC; 0.45 μm) and injected automatically into the HPLC system using an autosampler. Furthermore, detection was carried out at 280 nm. According to the obtained surface area of the peaks, depending on the concentration standard, the calibration curve for each standard was constructed. Quantification was based on external standards calibration. The linearity range and limit of quantification (LoQ) for each compound were: gallic acid (0.060–17.0 µg/mL; LoQ = 0.060 µg/mL), caffeic acid (0.010–17.0 µg/mL; LoQ = 0.211 µg/mL), epicatechin (0.250–50.0 µg/mL; LoQ = 0.250 µg/mL), rosmarinic acid (0.010–20.0 µg/mL; LoQ = 0.074 µg/mL), luteolin (0.003–7.2 µg/mL; LoQ = 0.087 µg/mL) and quercetin (0.050–20.0 µg/mL; LoQ = 0.131 µg/mL). Taking into account the obtained equation of linear concentration dependence, spectra, retention time and peak area, the concentrations of particular polyphenolic compounds in the tested samples were calculated, and the results were expressed as mg of compound per 100 g of sample.

### 3.6. In Vitro Antioxidant Activity

#### 3.6.1. Scavenging Capacity toward DPPH Radicals

Antioxidant activity towards DPPH radicals was determined by a spectrophotometric method [47]. First, 100 μL of examined extract solutions in series of different concentrations were prepared and then added to 2900 μL of DPPH methanolic solution (26 mg/L). After 1 h, absorbances were recorded at a wavelength of 517 nm. All experiments were performed in triplicate, and the mean values of the antioxidant potential were presented as mg of Trolox equivalents (TE) per g DW.

#### 3.6.2. Reducing Capacity of Fe^3+^ Ions

The reducing power of extracts was determined by the FRAP assay [48]. First, the FRAP reagent was prepared by mixing 10 mmol/L TPTZ in 40 mmol/L HCl, 20 mmol/L FeCl_3_, and acetate buffer, pH 3.6, in a ratio of 1:1:10, respectively. One hundred microliters of examined extract solutions in series of different concentrations were prepared, and 2900 µL of FRAP reagent was added. After 10 min in the dark at 37 °C, the absorbances were recorded at a wavelength of 593 nm. All samples were made in triplicate, and mean values of reducing power were presented as mg of Fe^2+^ per g DW.

#### 3.6.3. Scavenging Capacity towards ABTS^+^ Radicals

For the determination of antioxidant activity of *Thymus* extracts, ABTS assay was used as well [49]. ABTS stock solution was prepared from a mixture (1:1, *v/v*) of 2.45 mM potassium persulfate aqueous solution and 7 mmol ABTS (2,2′-azino-bis-(3-ethylbenzothiazoline-6-sulfonic acid) diammonium salt) aqueous solution and left in the dark at room temperature for 16 h. A stock solution was diluted using acetate buffer (pH 3.6) to an absorbance of 0.70 (±0.02) at wavelength of 734 nm. One hundred microliters of examined extract solutions in series of different concentrations were prepared and mixed with 2900 µL of ABTS reagent, after which they were stored in the dark at room temperature for 5 h. All samples were made in triplicate, and mean values of antioxidant activity were presented as mg of Trolox equivalents (TE) per g DW.

### 3.7. Nuclear Magnetic Resonance

NMR spectroscopy was performed to obtain 1D and 2D spectra (^1^H-NMR and ^1^H-^1^H NOESY) on a Bruker Avance III 400 spectrometer (Bruker, Billerica, MA, USA) at an operating frequency of 400.13 MHz according to the Meneses et al. [50] method. The NADES sample that was determined to be the best for polyphenol recovery (N16) was prepared in a 5 mm NMR tube. For the sample with NADES, 350 μL of NADES and 200 μL of DMSO-*d*_6_, and for pure components, approximately 5 mg of compound was added in the NMR tube and 500 μL of DMSO-*d*_6_. Chemical shifts were referenced to Me_4_Si (δ in ppm) and the data analysis was performed with MestReNova software (Bruker, Billerica, MA, USA) (11.0.4–18998).

### 3.8. Statistical Analysis

All experiments were performed in triplicate, and the results were expressed as the mean ± standard deviation (SD). All data from the screening of extraction solvent were analyzed by a one-factor-at-a-time (OFAT) approach using analysis of variance (ANOVA) with Tukey’s multiple comparison test at *p* < 0.05. Statistica 10.0 (StatSoft, Inc., Tulsa, OK, USA). Preliminary and optimization studies were analyzed by the Design of Experiments (DoE) using the aforementioned experimental designs. For multiple linear regression analysis, Design-Expert v.11 software (Stat-Ease, Minneapolis, MN, USA) was used. The goodness of fit was determined by ANOVA, while model adequacy was evaluated by the coefficient of determination (*R*^2^), coefficient of variance (CV) and *p*-values for the model and lack of fit. In order to verify the obtained empirical models, validation was performed by using the extracts prepared under optimized NADES conditions.

## 4. Conclusions

Wild thyme herbal dust has been efficiently utilized as a raw material for the recovery of polyphenolic antioxidants using NADES extraction. A three-step optimization approach was applied in order to maximize polyphenol content (TP and TF) and improve antioxidant activity determined by DPPH, FRAP and ABTS assays. Screening of the extraction solvent was initially performed by applying the 20 different NADES systems, while all other extraction parameters were held constant. l-proline-based solvents (N15 and N16) provided the highest TP and DPPH, and they were selected for further experiments. Preliminary experiments organized via 2^5−1^ fractional factorial design provided information about the most influential NADES extraction parameters and their experimental domain, which should be set in further steps. Finally, RSM with three factors (temperature, extraction time and L/S ratio) was applied for influence analysis and process optimization. Temperature and L/S ratio were the most impactful extraction parameters affecting polyphenol content and antioxidant activity. Multi-response optimization suggested a temperature of 65 °C, time of extraction of 180 min and L/S ratio of 28 g NADES/g DW were optimal set of extraction parameters. Experimental validation confirmed good agreement between predicted and experimentally observed data and NMR analysis suggested that interactions occurred probably in -OH and -NH groups from glycerol and l-proline in the most suitable NADES for polyphenol recovery (N16; Pro–Gly–H_2_O; 1:2:1). A literature comparison suggested that NADES extraction provides a tremendous upgrade in polyphenol content and antioxidant activity of wild thyme compared to emerging extraction techniques, such as UAE and PLE. Therefore, *T. serpyllum* herbal dust was valorized as a good underutilized sample for polyphenol recovery, and further research should be aimed toward the incorporation of wild thyme extracts obtained by NADES extraction in functional beverages.

## Figures and Tables

**Table 1:** 2^5−1^ fractional factorial design was used for the preliminary study with the experimental domain of the independent factors and observed values of target responses.

Run	Factors										Responses	
	A: Temperature [°C]		B: Extraction Time [min]		C: L/S Ratio [g NADES/g DW]		D: NADES Type *		E: Water Content [%]		TP [mg GAE/g]	DPPH [mg TE/g]
1	−1	50	1	120	1	20	Level 2	N16	−1	20	57.79	74.55
2	1	60	−1	60	1	20	Level 2	N16	−1	20	63.41	84.86
3	−1	50	1	120	−1	10	Level 2	N16	1	25	53.57	63.45
4	−1	50	1	120	1	20	Level 1	N15	1	25	54.19	57.96
5	−1	50	−1	60	1	20	Level 1	N15	−1	20	50.62	54.25
6	1	60	−1	60	1	20	Level 1	N15	1	25	52.11	52.50
7	1	60	1	120	−1	10	Level 1	N15	1	25	44.13	37.59
8	1	60	1	120	−1	10	Level 2	N16	−1	20	59.52	69.75
9	−1	50	−1	60	−1	10	Level 2	N16	−1	20	51.87	54.60
10	1	60	1	120	1	20	Level 1	N15	−1	20	52.37	54.25
11	−1	50	−1	60	1	20	Level 2	N16	1	25	58.47	64.19
12	1	60	1	120	1	20	Level 2	N16	1	25	64.24	91.16
13	−1	50	−1	60	−1	10	Level 1	N15	1	25	43.31	39.97
14	1	60	−1	60	−1	10	Level 2	N16	1	25	54.84	72.47
15	−1	50	1	120	−1	10	Level 1	N15	−1	20	44.45	40.13
16	1	60	−1	60	−1	10	Level 1	N15	−1	20	44.93	39.88

* categorical variable.

**Table 2:** Face-centered central composite experimental design with real and coded NADES extraction parameters and experimentally obtained values of investigated target responses (TP, TF, DPPH, FRAP and ABTS).

Run	Factor 1		Factor 2		Factor 3		Response 1	Response 2	Response 3	Response 4	Response 5
	A: Temperature [°C]		B: Extraction Time [min]		C: L/S Ratio [g NADES/g DW]		TP [mg GAE/g]	TF [mg CE/g]	DPPH [mg TE/g]	FRAP [mg Fe^2+^/g]	ABTS [mg TE/g]
1	1	70	1	180	−1	10	59.69	4.82	110.59	37.63	125.42
2	−1	40	−1	60	−1	10	49.13	10.29	78.63	19.85	70.81
3	0	55	0	120	0	20	61.64	23.48	143.49	42.03	140.84
4	0	55	1	180	0	20	65.69	22.29	145.81	33.75	120.62
5	0	55	0	120	0	20	62.53	23.54	144.10	33.29	116.87
6	0	55	0	120	0	20	59.14	23.48	145.61	33.19	115.34
7	0	55	0	120	1	30	66.74	20.55	174.14	35.56	113.90
8	0	55	0	120	0	20	59.34	23.02	147.25	42.61	139.94
9	−1	40	1	180	1	30	54.86	24.00	156.07	25.62	84.75
10	0	55	0	120	0	20	59.61	23.22	152.76	44.70	147.87
11	−1	40	1	180	−1	10	47.38	10.37	89.46	21.56	75.29
12	1	70	0	120	0	20	70.01	22.70	155.44	37.51	118.08
13	1	70	−1	60	1	30	67.74	20.33	188.08	38.12	119.57
14	−1	40	−1	60	1	30	53.82	24.66	153.19	24.09	77.97
15	1	70	−1	60	−1	10	57.65	6.01	125.61	33.17	100.35
16	0	55	0	120	−1	10	53.40	5.20	120.33	33.38	100.96
17	1	70	1	180	1	30	71.43	19.34	188.01	42.00	124.55
18	−1	40	0	120	0	20	48.27	14.37	116.73	23.29	77.56
19	0	55	−1	60	0	20	57.82	24.65	143.44	34.08	98.24

**Table 3:** Analysis of variance (ANOVA) and descriptive statistics (R^2^ and CV) of the fitted model for all investigated responses (TP, TF, DPPH, FRAP and ABTS).

Source	Sum of	df *	Mean	F-Value	*p*-Value
**TP**					
Model	831.71	9	92.41	11.7975	0.00055
Residual	70.50	9	7.83		
Lack of Fit	61.07	5	12.21	5.1803	0.06809
Pure Error	9.43	4	2.36		
Cor Total	902.21	18			
R^2^	0.922				
CV [%]	4.72				
**TF**					
Model	844.22	9	93.80	9.4944	0.00127
Residual	88.92	9	9.88		
Lack of Fit	88.72	5	17.74	359.7893	<0.0001
Pure Error	0.20	4	0.05		
Cor Total	933.14	18			
R^2^	0.905				
CV [%]	17.24				
**DPPH**					
Model	14,833.37	9	1648.15	61.0444	<0.0001
Residual	242.99	9	27.00		
Lack of Fit	187.76	5	37.55	2.71975	0.17690
Pure Error	55.23	4	13.81		
Cor Total	15,076.36	18			
R^2^	0.984				
CV [%]	3.69				
**FRAP**					
Model	829.49	9	92.17	4.9684	0.01280
Residual	166.95	9	18.55		
Lack of Fit	45.98	5	9.20	0.3041	0.88809
Pure Error	120.97	4	30.24		
Cor Total	996.45	18			
R^2^	0.832				
CV [%]	12.88				
**ABTS**					
Model	7899.47	9	877.72	4.0763	0.02406
Residual	1937.89	9	215.32		
Lack of Fit	1038.55	5	207.71	0.9238	0.54581
Pure Error	899.34	4	224.84		
Cor Total	9837.36	18			
R^2^	0.803				
CV [%]	13.48				

* degrees of freedom.

**Table 4:** Experimental validation of RSM optimization for NADES extraction of polyphenols and antioxidants from wild thyme.

Input and Output Parameters	Goal	Lower Limit	Upper Limit	Predicted Values	Experimental Values
				Optimal Conditions	Optimal Conditions
Temperature [°C]	is in range	40	70	65	65
Extraction time [min]	is in range	60	180	180	180
L/S ratio [g NADES/g DW]	is in range	10	30	28	28
TP [mg GAE/g]	maximize	43.38	71.43	71.43	71.43 ± 1.17
TF [mg CE/g]	maximize	4.82	24.66	22.81	19.43 ± 0.20
DPPH [mg TE/g]	maximize	78.63	188.08	179.52	188.01 ± 11.19
FRAP [mg Fe^2+^/g]	maximize	19.85	44.70	41.09	42.00 ± 0.28
ABTS [mg TE/g]	maximize	70.80	147.87	130.06	124.55 ± 3.25

**Table 5:** Content of major polyphenols (phenolic acids and flavonoids) in wild thyme extract obtained under optimal conditions.

No.	Compound	Content [mg/100 g]
1.	Gallic acid	20.61
2.	Caffeic acid	25.83
3.	Epicatechin	21.06
4.	Rosmarinic acid	524.18
5.	Luteolin	28.27
6.	Quercetin	42.27

**Table 6:** Chemical content of applied NADES mixtures in the screening of extraction solvent.

Code	Content	Molar Ratio	Water Content [%]
N1	Citric acid (CA)–glucose (Glu)	1:1	-
N2	Citric acid (CA)–sucrose (Suc)	1:1	-
N3	Citric acid (CA)–betaine (Bet)–water (H_2_O)	1:1:1	5.50
N4	Choline chloride (ChCl)–glucose (Glu)	1:1	-
N5	Glycerin (Gly)–betaine (BET)	2:1	-
N6	Betaine (Bet)–glycerine (Gly)–water (H_2_O)	1:2:1	5.64
N7	Betaine (Bet)–glucose (Glu)	1:1	-
N8	Glycerin (Gly)–fructose (Fru)	4:1	-
N9	Choline chloride (ChCl)–glycerin (Gly)	1:2	-
N10	Choline chloride (ChCl)–glycerin (Gly)–water (H_2_O)	2:1:1	5.27
N11	Lactic acid (LA)–glucos –water (H_2_O)	5:1:3	7.89
N12	Choline chloride (ChCl)–lactic acid (LA)	1:4	11.23
N13	Glucose (Glu)–tartaric acid (TA)	1:1	-
N14	Lactic acid (LA)–fructose (Fru)	5:1	11.16
N15	l-proline (Pro)–lactic acid (LA)	1:2	9.69
N16	l-proline (Pro)–glycerin (Gly)–water (H_2_O)	1:2:1	5.68
N17	Malic acid (MA)–betaine (Bet)–water (H_2_O)	2:1:5	18.95
N18	Tartaric acid (TA)–betaine (Bet)–water (H_2_O)	2:1:5	17.75
N19	Choline chloride (ChCl)–citric acid (CA)	1:1	-
N20	1,2-Propanediol (PD)–choline chloride (ChCl)–water (H_2_O)	1:1:1	7.71

**Table 7:** Experimental plan for the isolation of polyphenolic antioxidants from wild thyme.

**I Step—Screening of the Extraction Solvent**			
**Approach**	**Constant Parameters**	**Factors**	Responses *
OFAT ^1^	Sample to solvent ratio: 1:20 m/mTemperature: 50 °C Extraction time: 60 min Stirring speed: 700 rpm Water content: 20%	NADES: N1–N20	TP ^2^ DPPH ^3^
**II Step—Preliminary Study**			
**Approach**	**Constant Parameters**	**Factors**	**Responses**
2^5−1^ fractional factorial design	Stirring speed: 700 rpm	Temperature: 50 and 60 °C Extraction time: 60 and 120 min Sample to solvent ratio: 1:10 and 1:20 m/m NADES: N15 and N16 Water content: 20 and 25%	TP DPPH
**III Step—Optimization**			
**Approach**	**Constant Parameters**	**Factors**	**Responses**
RSM	NADES: N16 Water content: 20% Stirring speed: 700 rpm	Temperature: 40, 55 and 70 °C Extraction time: 60, 120 and 180 min Sample to solvent ratio: 1:10, 1:20 and 1:30 m/m	TP TF ^4^ DPPH FRAP ^5^ ABTS ^6^

* maximized, ^1^ One-factor-at-a-time, ^2^ total phenol content, ^3^ antioxidant activity toward DPPH radicals, ^4^ total flavonoids content, ^5^ reducing activity towards Fe^3+^ ions, ^6^ antioxidant activity towards ABTS+ radicals.

## Data Availability

Data are contained within the article or Supplementary Material.

## Supplementary Materials

The following supporting information can be downloaded, Figure S1. Influence of NADES extraction factors on (a) TP and (b) DPPH obtained in preliminary experiments; Table S1. Significance of linear, cross product and quadratic terms on TP, TF, DPPH, FRAP and ABTS; Figure S2. The effect of NADES extraction parameters (temperature, extraction time and L/S ratio) on antioxidant activity determined by (a) DPPH, (b) FRAP and (c) ABTS assays.

## Sample Availability

Samples of the compounds are not available from the authors.
