# Supplementary material for: Natural Deep Eutectic Solvent (NADES) Extraction Improves Polyphenol Yield and Antioxidant Activity of Wild Thyme (Thymus serpyllum L.) Extracts
Source: Molecules. 2022 Feb 23;27(5):1508. doi: 10.3390/molecules27051508 (PMC8911718; doi:10.3390/molecules27051508)
Supplement: Supplementary file 1 [file molecules-27-01508-s001.zip › molecules-1590974-supplementary.pdf]

molecules

MDPI

Supplementary Material

Natural Deep Eutectic Solvent (NADES) Extraction Improves Polyphenol Yield and Antioxidant Activity of Wild Thyme (*Thymus serpyllum* L.) Extracts

Branimir Pavlić [1,*], Živan Mrkonjić [1], Nemanja Teslić [2], Aleksandra Cvetanović Kljakić [1], Milica Pojić [2], Anamarija Mandić [2], Alena Stupar [2], Filipa Santos [3], Ana Rita C. Duarte [3] and Aleksandra Mišan [2,*]

[1] Faculty of Technology, University of Novi Sad, Blvd. cara Lazara 1, 21000 Novi Sad, Serbia; zivan_mrkonjic@hotmail.com (Ž.M.); a.c.istrazivac@gmail.com (A.C.K.)

[2] Institute of Food Technology, University of Novi Sad, Blvd. cara Lazara 1, 21000 Novi Sad, Serbia; nemanja.teslic@fins.uns.ac.rs (N.T.); milica.pojic@fins.uns.ac.rs (M.P.); anamarija.mandic@fins.uns.ac.rs (A.M.); alena.tomsik@fins.uns.ac.rs (A.S.)

[3] LAQV, REQUIMTE, Departamento de Química, Nova School of Science and Technology, 2829-516 Caparica, Portugal; mfca.santos@campus.fct.unl.pt (F.S.); ard08968@fct.unl.pt (A.R.C.D.)

* Correspondence: bpavlic@uns.ac.rs (B.P.); aleksandra.misan@fins.uns.ac.rs (A.M.)

a)

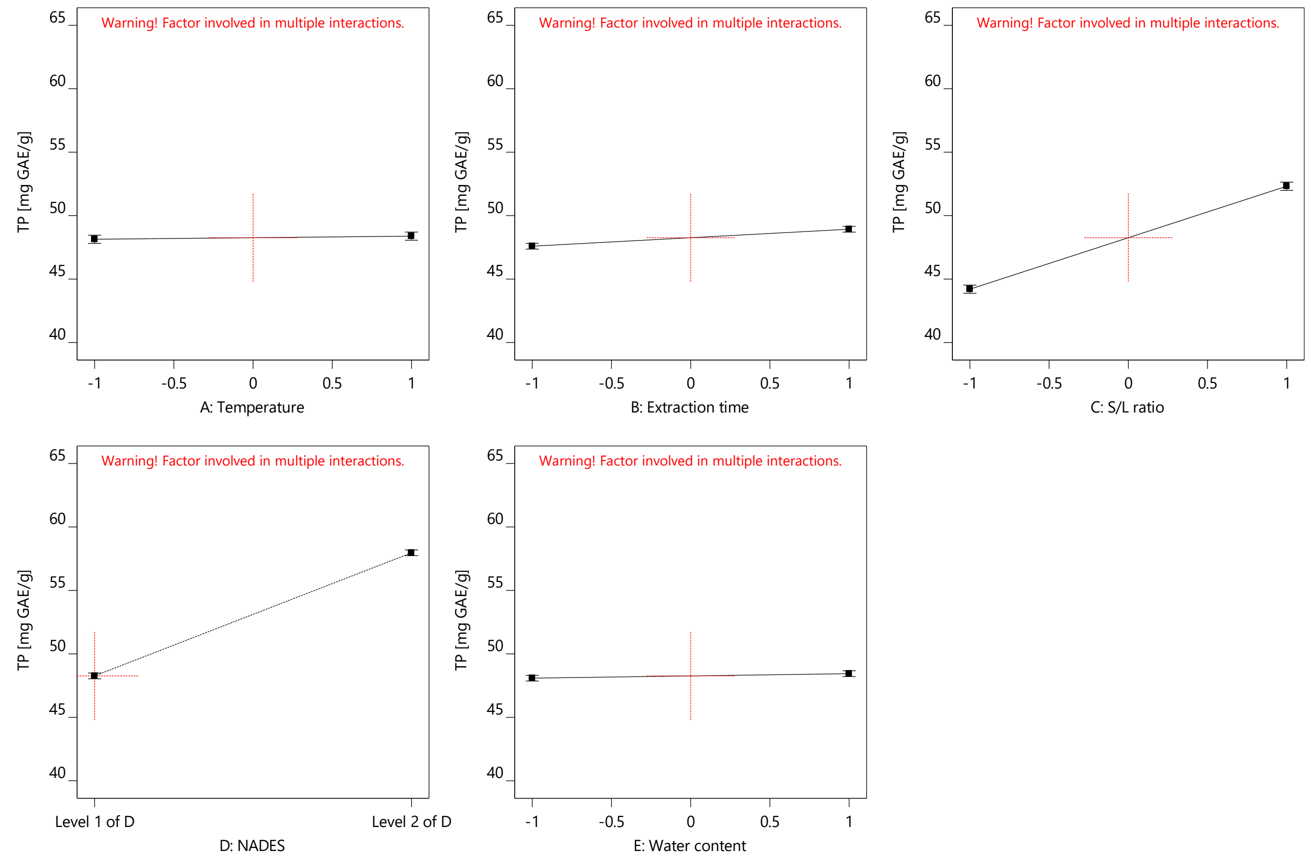

b)

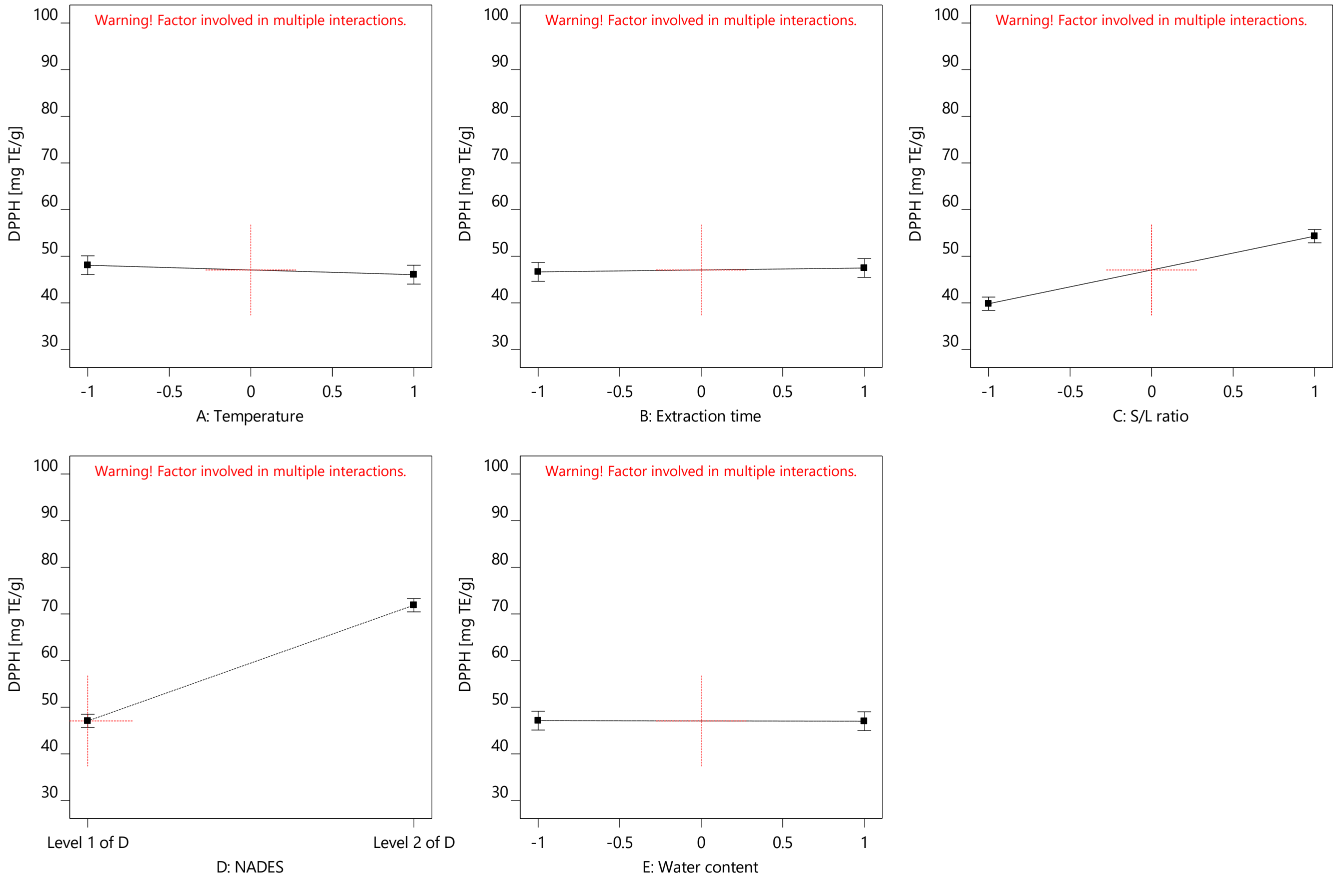

Figure S1. Influence of NADES extraction factors on a) TP and b) DPPH obtained in preliminary experiments.

Table S1. Significance of linear, cross product and quadratic terms on TP, TF, DPPH, FRAP and ABTS.

Term	TP	TF	DPPH	FRAP	ABTS
A-Temperature	<0.0001*	0.3181	<0.0001*	0.0004*	0.00187*
B-Extraction time	0.1789	0.6194	0.9537	0.4304	0.20327
C-L/S ratio	0.0005*	0.0000*	0.0000*	0.1800	0.32863
AB	0.4365	0.8610	0.0818	0.6850	0.66156
AC	0.2531	0.9264	0.9330	0.9351	0.96766
BC	0.5886	0.9529	0.6450	0.9520	0.67810
A^2	0.2286	0.2590	0.0112*	0.0805**	0.08920**
B^2	0.8031	0.1981	0.6569	0.5516	0.56540
C^2	0.4777	0.0024*	0.7188	0.6950	0.43238

*$p < 0.05$—significant, **$0.05 < p < 0.1$—moderately significant.

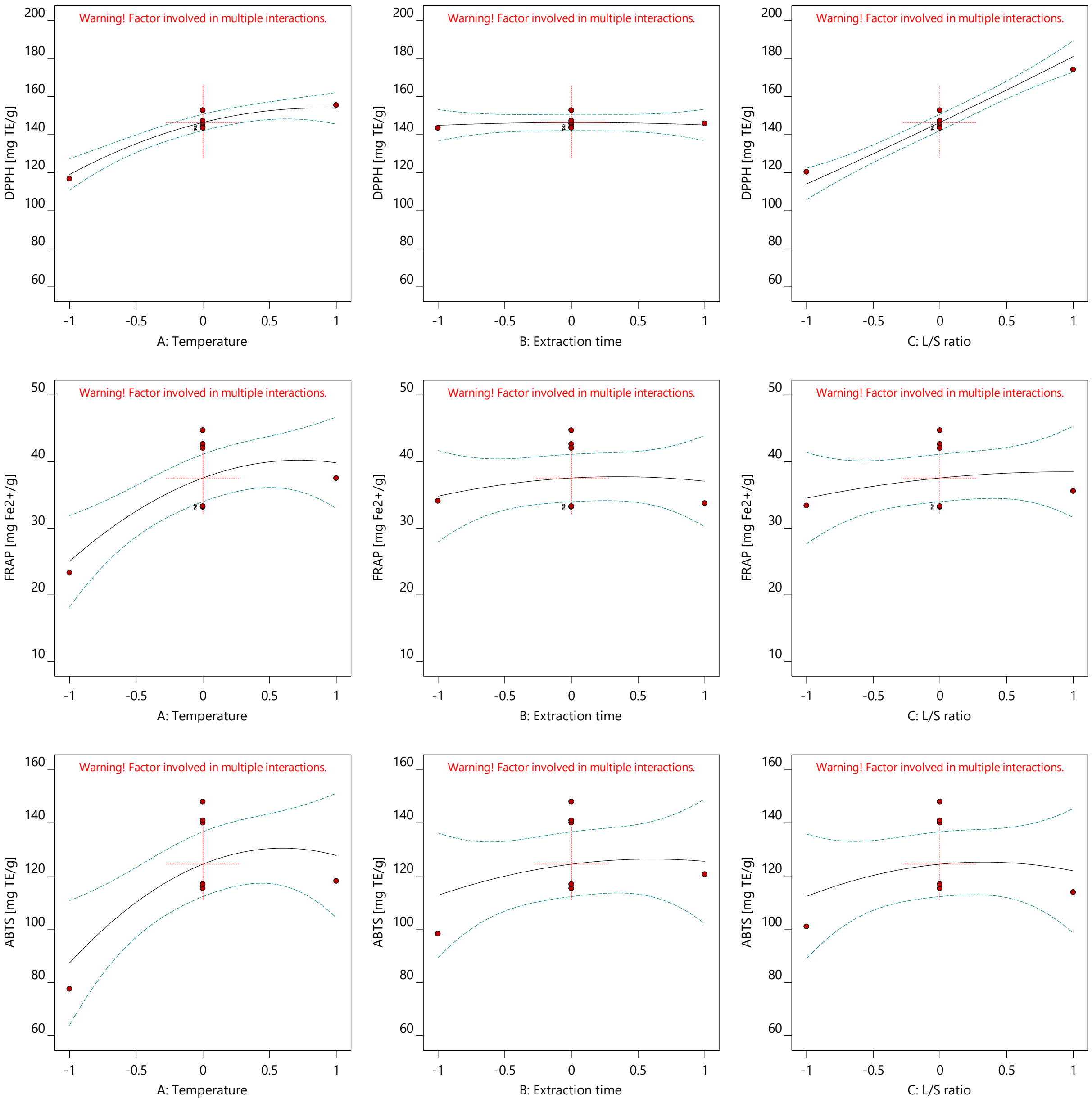

Figure S2. The effect of NADES extraction parameters (temperature, extraction time and L/S ratio) on antioxidant activity determined by a) DPPH, b) FRAP and c) ABTS assays.
